# Post-surgical infection in elective cardiac surgeries: what is the role of dental evaluation? A retrospective study

**DOI:** 10.31744/einstein_journal/2026AO2495

**Published:** 2026-07-29

**Authors:** Ana Eliza Durães de Faria, Itamara Lúcia Itagiba Neves, Ricardo Simões Neves, Samara Caroline Fernandes Galvani, Henrique Furukawa, Bianca de Fatima Borim Pulino, Heloisa Fonseca Marão, Raphael Capelli Guerra, Marcela Alves dos Santos-Paul

**Affiliations:** 1 Universidade de São Paulo São Pulo SP Brazil Universidade de São Paulo, São Pulo, SP, Brazil.; 2 Universidade Estadual de Campinas Faculdade de Odontologia de Piracicaba Piracicaba SP Brazil Faculdade de Odontologia de Piracicaba, Universidade Estadual de Campinas, Piracicaba, SP, Brazil.; 3 Hospital Israelita Albert Einstein Instituto Israelita de Ensino e Pesquisa Albert Einstein São Pulo SP Brazil Instituto Israelita de Ensino e Pesquisa Albert Einstein, Hospital Israelita Albert Einstein, São Pulo, SP, Brazil.; 4 Instituto Sirio-Libanes de Ensino e Pesquisa Hospital Sírio-Libanês São Pulo SP Brazil Instituto Sirio-Libanes de Ensino e Pesquisa, Hospital Sírio-Libanês, São Pulo, SP, Brazil.; 5 Universidade de Santo Amaro São Pulo SP Brazil Universidade de Santo Amaro, São Pulo, SP, Brazil.

**Keywords:** Infections, Cardiovascular surgical procedures, Thoracic surgical procedures, Diagnosis, oral, Dental care, Oral health, Risk factors

## Abstract

**Objective:**

To evaluate the prevalence and clinical characteristics of post-surgical infections after coronary and/or valvular heart surgery at a tertiary hospital and to examine the relationship between these infections and prior dental evaluation.

**Methods:**

A retrospective study was conducted among patients who underwent elective cardiac surgery at InCor-HCFMUSP between January 2018 and April 2019.

**Results:**

The sample included 1,276 surgeries, of which 10.6% were associated with post-surgical infection. Length of hospital stay and infection-related mortality differed significantly between patients with and without post-surgical infection (p<0.0001). Surgical site infections were the most prevalent infections, accounting for 6.2% of the sample, whereas pneumonia was the least frequent, accounting for 0.2%. Dental evaluation was performed in 62.3% of patients in the Valve Surgery Group and 25.8% of those in the Coronary Surgery Group. Among patients who underwent dental evaluation, 89.8% did not develop infection; dental evaluation was also significantly associated with length of hospital stay in the Valve and Coronary Surgery Groups (p=0.0187 and p=0.0317, respectively).

**Conclusion:**

Post-surgical infection occurred in 10.6% of surgeries. Prior dental evaluation was not significantly associated with post-surgical infection, although a favorable percentage trend was observed among evaluated patients. Poor oral health is a risk factor for certain infections; however, these infections were not frequent in this sample. Hospital stay was shorter among patients who underwent dental evaluation.

## Highlights

The post-surgical infection rate after elective cardiac surgery was 10.6%.

Preoperative dental assessment was not significantly associated with reduced post-surgical infection.

Preoperative dental assessment was associated with a shorter hospital stay (p<0.05).

Overall, 89.8% of evaluated patients remained infection-free, indicating a favorable trend.

## In Brief

A retrospective study of 1,276 surgeries found that preoperative dental assessment was not significantly associated with reduced post-surgical infections (10.6%) but was associated with a shorter hospital stay (p < 0.05). Among evaluated patients, 89.8% remained infection-free, indicating a favorable trend and a potential clinical and economic benefit.

## Graphical abstract

**Figure:**
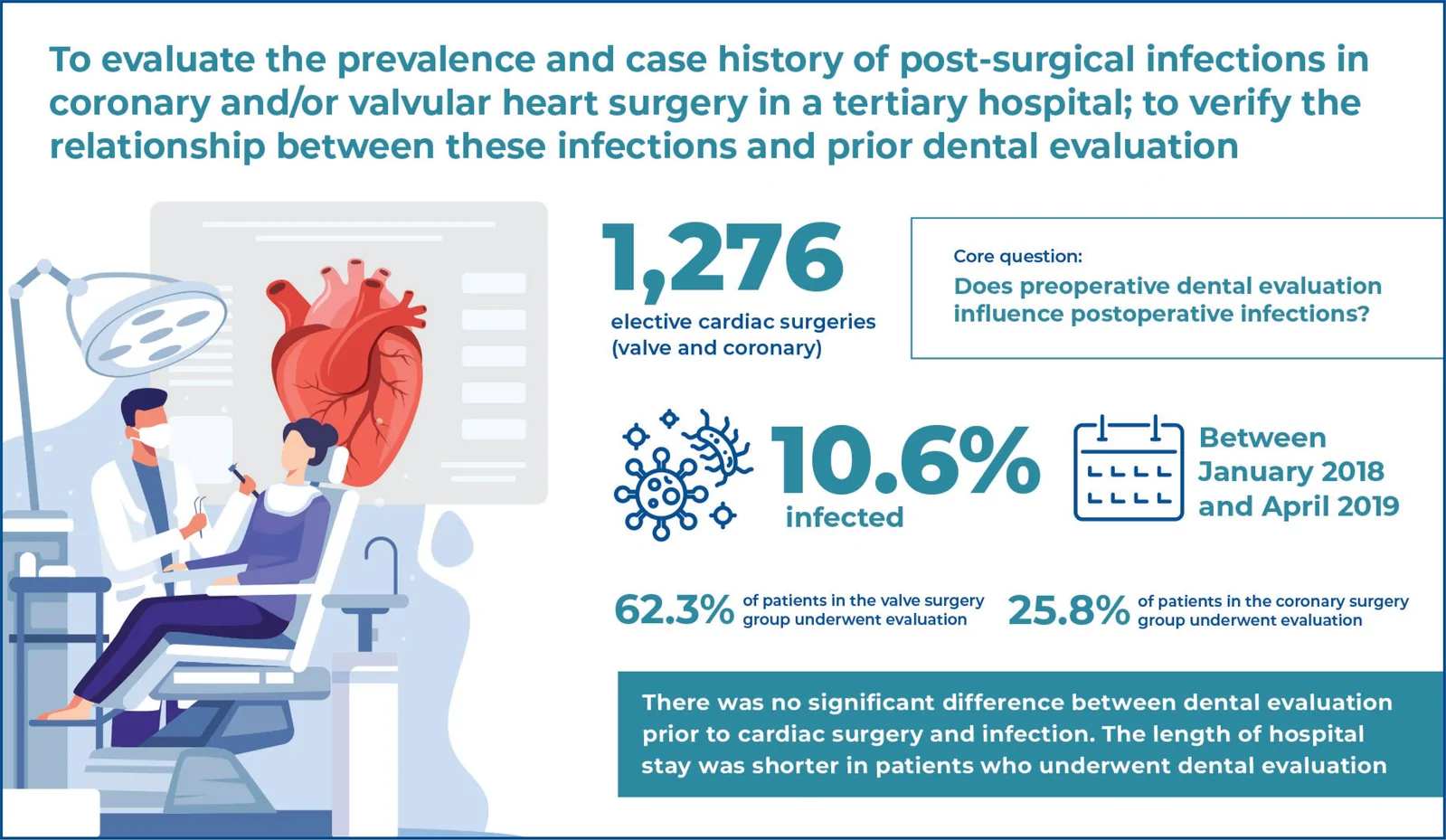

## INTRODUCTION

Cardiac surgery aims to prolong patients’ lives and reduce cardiovascular disease-related morbidity. Despite significant advances in these procedures, postoperative complications, including infections, remain important non-cardiac complications and are associated with increased morbidity, mortality, length of hospital stay, and healthcare-resource use.^(1–4)^ Preoperative patient preparation supports perioperative stability, helps prevent complications, and promotes favorable postoperative outcomes.^(1,5)^

Postoperative infections may include surgical site infection, mediastinitis, urinary tract infection, primary bloodstream infection, gastrointestinal infection, pulmonary infection, and endocardial infection, with manifestations ranging from local inflammation and superficial wound infection to severe septicemia.^(1,3)^ Several risk factors are associated with healthcare-associated infections (HAIs), including age, nutritional status, diabetes mellitus, smoking, obesity, coexisting infections at other sites, length of hospital stay, mechanical ventilation, and breaches of asepsis.^(5,6)^

The oral cavity contains numerous microorganisms; therefore, invasive dental procedures, oral hygiene, and chewing can cause transient bacteremia.^(7)^ In patients with altered immune responses or sites susceptible to infection, bacteremia may be a risk factor.^(8)^ Although the biological mechanisms are not fully understood, oral microbiota homeostasis is closely related to health status.^(9)^ Some studies^(10,11)^ have associated pulmonary infections and endocarditis with oral health; however, whether intraoral infectious foci before elective cardiac surgery influence the development of other post-surgical infections (PSIs) remains unclear.

To date, published guidelines have not provided conclusive information on key issues related to dental treatment for patients preparing for cardiac surgery, including which treatments should be performed before surgery, the optimal timing of dental treatment, and the risks and benefits of dental treatment before cardiovascular procedures.^(9,12)^ Several studies^(13–18)^ have evaluated the influence of dental treatment and oral health on cardiac surgery outcomes, and dental treatment has been considered beneficial for patients with cardiovascular disease, particularly those undergoing valve surgery or heart transplantation.^(18)^

At the Heart Institute of the *Hospital das Clínicas, Faculdade de Medicina, Universidade de São Paulo* (InCor-HCFMUSP), the Valve Surgery Group (VSG) routinely recommends dental evaluation as part of the preoperative assessment for all patients, whereas the Coronary Surgery Group (CSG) requests preoperative dental evaluation only in selected cases, such as suspected dental abscess or severe coronary artery disease. Thus, the InCor Dentistry Unit prepares patients scheduled for cardiac surgery by identifying and treating oral infectious foci. The protocol is based on detailed clinical and radiographic evaluation while considering each patient's systemic condition. Oral infectious foci may include carious lesions involving dentin, supra- and subgingival calculus, radiographically detected periapical lesions with or without signs of inflammation, residual roots, dentoalveolar or periodontal abscesses or fistulas, moderate to severe periodontal disease, teeth requiring endodontic treatment, and intraoral candidiasis. Dental clearance requires treatment of all identified infectious foci.

## OBJECTIVE

Therefore, this study aimed to evaluate the prevalence and clinical characteristics of post-surgical infections after elective coronary and/or valvular heart surgery at InCor and to examine the relationship between these infections and prior dental assessment before cardiac surgery.

## METHODS

This retrospective study evaluated the prevalence of post-surgical infection after elective cardiac surgery at InCor-HCFMUSP between January 2018 and April 2019. The study was approved by the Ethics Committee of *Hospital das Clínicas, Faculdade de Medicina, Universidade de São Paulo* (CAAE: 27224619.5.0000.0068; # 3.807.023).

### Data sources

Data were collected from the institution's database. The following variables were collected: date of birth; date of admission; date of surgery; date of discharge; date of infection; date of dental care at the InCor Dental Unit; patient registration number; name; sex; age; surgical procedure; outcome at discharge (discharge or death); patient group (valvular heart disease, congenital heart disease, coronary artery disease, aortic disease, or other cardiac surgery); surgical indication (elective, emergency, urgent, or salvage); infection classification (post-surgical, admission-onset, or hospital-acquired); diagnosis; and microorganism identified. Electronic medical records (Institutional System - Si3) were also reviewed to obtain additional information, including pre-existing non-cardiac comorbidities and any history of prior coronary artery bypass grafting (CABG).

### Eligibility criteria

Data from patients who underwent elective coronary and/or valve surgery between January 2018 and April 2019 were included. Data from urgent, emergency, and/or salvage surgeries, hospital-acquired and/or admission-onset infections, and medical records with incomplete information were excluded.

### Definitions

Surgical indication was defined as follows: urgent surgery required intervention earlier than planned. Emergency surgery required immediate intervention because of imminent risk to life. Salvage surgery required cardiopulmonary resuscitation by external cardiac massage on the way to the operating room or before induction of anesthesia, representing a more extreme situation than emergency surgery. Elective surgeries were scheduled procedures.^(19)^

Definitions of dental assessment and dental clearance: For the purposes of this study, dental assessment (DA) was defined as the performance of a clinical and radiographic examination (panoramic and/or periapical) by a dentist from the InCor team, regardless of whether treatment was completed. Dental clearance was defined as the status assigned to a patient who, after DA and treatment of all identified infectious foci—caries involving dentin, supra- and subgingival calculus, periapical lesions, residual roots, abscesses, fistulas, moderate-to-severe periodontal disease, and intraoral candidiasis—was discharged by the dental team without active infectious foci that would contraindicate the surgical procedure. Patients who did not undergo DA or did not complete treatment were classified as "no assessment" or "no clearance," as appropriate.

Healthcare-associated infections (HAIs) at the institution are monitored by the Hospital Infection Control Committee (CCIH) according to the guidelines of the Brazilian National Health Surveillance Agency (ANVISA — *Agência Nacional de Vigilância Sanitária*). Diagnostic criteria for HAIs are based on epidemiological data, and infections are classified as: post-surgical (occurring within 30 days after surgery, or within 90 days if an implant was placed or deep soft tissues were involved, under specific conditions); hospital-acquired (occurring more than 48 hours after admission); and admission-onset (diagnosed before or within 48 hours of admission, with no prior surgical procedure).^(20)^

Date of infection was defined as the date on which the first sign, symptom, or diagnostic test used to define the infection occurred within the 7-day infection window period.^(20)^

### Data analysis

Data are presented as frequencies, percentages, means, and standard deviations. Categorical variables were compared using the chi-square test or Fisher's exact test (when an expected frequency was less than 5), and continuous variables were compared using Student's *t* test. All analyses were performed using R software, with the significance level set at 5%.

## RESULTS

A total of 1,886 surgeries were performed in 1,873 patients. Of these, 607 surgeries were excluded because they did not meet the inclusion criteria, leaving 1,279 surgeries in 1,274 patients. Patients were divided into two groups: the CSG, with 507 surgeries (39.6%), and the VSG, with 772 surgeries (60.4%). Patients who underwent both valve surgery and coronary artery bypass grafting in the same procedure were allocated to the VSG. Post-surgical infection occurred in 137 cases (10.6%), including 52 (4.0%) in the CSG and 85 (6.6%) in the VSG (Figure 1). No significant association was observed between infection status and type of surgery performed (p=0.5089) (Table 1).

**Figure 1:**
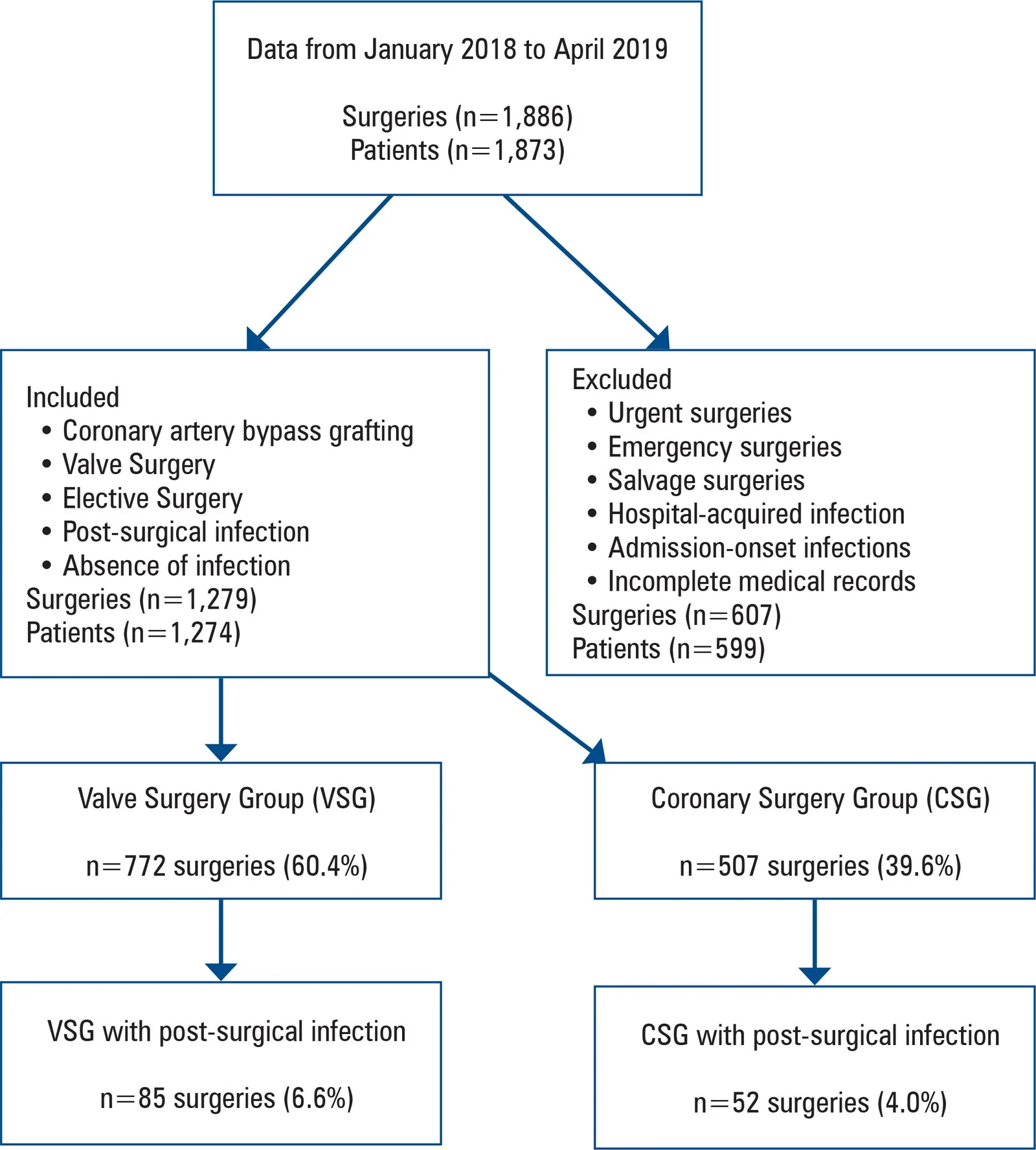
Flowchart of patient selection for the study

**Table 1:** Results by surgical group

Variable	Category	n (%)	Type of surgery		p value
			Valve	Coronary	
			n (%)		
Infection	No infection	1142 (89.2)	687 (89)	455 (89.7)	0.5089
	With infection	137 (10.8)	85 (11)	52 (10.3)	
Sex	Male	716 (56.2)	328 (42.8)	388 (76.5)	<0.0001
	Female	558 (43.8)	439 (57.2)	119 (23.5)	
Discharge type	Death	78 (6.1)	51 (6.6)	27 (5.3)	0.3347
	Discharged	1201 (93.9)	721 (93.4)	480 (94.7)	

Of the total sample, 716 patients (56.2%) were male; of these, 388 (76.5%) were in the CSG. Among female patients, 439 (57.2%) were in the VSG. Sex distribution differed significantly between the groups (p<0.0001) (Table 1).

The mean age was 59.8±13.2 years and was significantly higher in the CSG than in the VSG (63.5±8.9 *versus* 57.4±14.9 years, respectively; p<0.0001). In both groups, the most frequent comorbidity was systemic arterial hypertension (SAH), followed by dyslipidemia (DLP) and diabetes mellitus (DM). In the VSG, 52.5% of patients had SAH, 23.2% had DLP, and 17.2% had DM. In the CSG, 86.6% of patients had SAH, 53.65% had DLP, and 49.9% had DM; comorbidities were significantly more frequent in the CSG than in the VSG (p<0.0001). No significant association was observed between infection and comorbidities in either the VSG (p=0.4750) or the CSG (p=0.6222).

### Length of stay and discharge type

The mean length of stay was 17±14 days in the VSG and 14±12 days in the CSG, with a significantly longer stay in the VSG (p<0.0001). In both groups, patients with post-surgical infection had a longer length of stay.

Discharge type did not differ significantly between the VSG and CSG (p=0.3347) (Table 1). In both groups, infection status was significantly associated with discharge type, with a higher proportion of deaths among patients with infection in the total sample (p<0.0001), VSG (p<0.0001), and CSG (p=0.007) (Figure 2).

**Figure 2:**
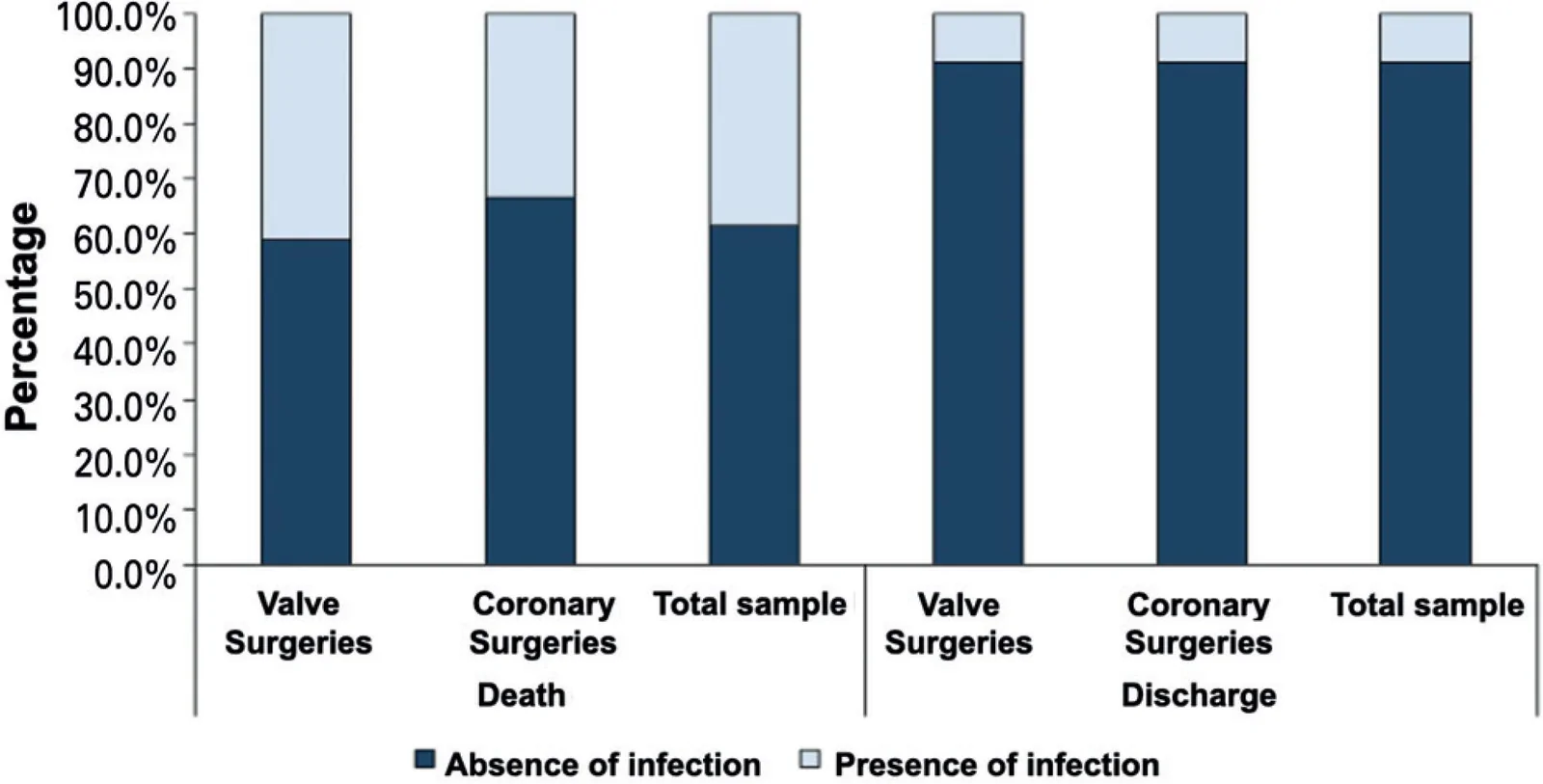
Percentage of patients with and without post-surgical infection according to discharge type

### Microorganisms and infection diagnoses

The most prevalent microorganism in post-surgical infection cases in both groups was Staphylococcus epidermidis, accounting for more than 20% of cases, followed by Klebsiella pneumoniae and Staphylococcus aureus (Table 2).

**Table 2:** Microorganisms involved in post-surgical infections by group

Groups		Valve Surgery Group*	Coronary Surgery Group#
		Infected Patients n (%)	Infected Patients n (%)
Microorganisms			
	*Staphylococcus epidermidis*	21 (247)	13 (25)
	*Klebsiella pneumoniae*	17 (200)	10 (192)
	*Staphylococcus aureus*	9 (106)	8 (154)
	*Escherichia coli*	6 (70)	4 (77)
	Others* #	32 (377)	17 (327)

* Other microorganisms in the VSG included C. glabrata, E. cloacae, S. marcescens, S. capitis, C. parapsilosis, C. tropicalis, C. freundii, F. magna, P. mirabilis, coagulase-negative Staphylococcus, T. maltophilia, S. gallolyticus (bovis), E. faecium, K. aerogenes, and P. aeruginosa.

# Other microorganisms in the CSG included S. marcescens, C. albicans, Corynebacterium sp., E. aerogenes, Enterobacter cloacae complex, C. dubliniensis, coagulase-negative Staphylococcus, S. anginosus, and S. viridans.

Deep incisional surgical site infections (SSIs) were the most prevalent infection diagnoses, whereas pneumonia was the least prevalent in both groups (Table 3).

**Table 3:** Prevalence of infection diagnoses by group

Groups	Valve Surgery Group	Coronary Surgery Group
Diagnosis of infection	Infected patients n (%)	Infected patients n (%)
Primary bloodstream infection	14 (16.5)	7 (13.5)
Deep incisional SSI	23 (27)	22 (42.3)
Superficial incisional SSI	7 (8.2)	8 (15.4)
SSI organ/space	16 (18.8)	3 (5.8)
Urinary tract infection*	16 (18.8)	8 (15.4)
Other lower respiratory tract infections#	7 (8.2)	3 (5.8)
Pneumonia	2 (2.3)	1 (1.9)
Total	85	52

* Includes asymptomatic bacterial infections;

# includes bronchitis, tracheobronchitis, tracheitis, and bronchiolitis.

### Dental assessment

Patients were classified into a dental clearance group, comprising those who underwent dental assessment and dental treatment before cardiac surgery and were therefore considered to have no intraoral infectious foci, and a no-assessment group, comprising patients who did not undergo prior dental assessment or treatment. The VSG had a significantly higher percentage of patients with dental clearance than the CSG (62.3% vs. 25.8%, respectively; p<0.0001). The group without dental assessment had a significantly higher mean age than the group with dental assessment (61.3±12.9 vs. 58.3±13.2 years, respectively; p<0.0001).

No significant association was observed between post-surgical infection and prior dental assessment (p=0.5276). However, descriptively, 10.2% of patients with dental assessment developed infection, whereas 89.8% did not develop infection (Table 4).

**Table 4:** Presence or absence of infection according to prior dental assessment

	Valve Surgery Group		Coronary Surgery Group		Total sample	
	Dental assessment n (%)	No dental assessment n (%)	Dental assessment n (%)	No dental assessment n (%)	Dental assessment n (%)	No dental assessment n (%)
With Infection	51 (10.7)*	34 (11.8)*	11 (8.4)*	41 (10.9)*	62 (10.2)*	75 (11.5)*
No Infection	430 (89.3)*	257 (88.2)*	120 (91.6)*	335 (89.1)*	574 (89.8)*	590 (88.7)*
Total	481 (62.3)#	291 (37.7)#	131 (25.8)#	376 (74.2)#	609 (47.8)#	665 (52.2)#
p value	0.6396		0.4153		0.5276	

* Column percentage;

# row percentage.

Because the InCor Dentistry Unit specializes in the care of cardiac patients, particularly those undergoing cardiac surgery, patients were reclassified by considering only those who underwent evaluation and treatment at the institution as eligible for dental clearance. The percentage of patients in this group was significantly higher in the VSG than in the CSG (23.5% *versus* 6.9%, respectively; p<0.0001), consistent with the previous analysis. No significant association was observed between InCor dental assessment and post-surgical infection (p=0.1395); descriptively, 7.9% of patients who underwent InCor dental assessment developed infection, whereas 92.1% did not (Tables 4 and 5). In the VSG and total sample, the p-values were closer to the threshold for statistical significance in this analysis.

**Table 5:** Presence or absence of infection according to prior oral healthcare at the InCor Dentistry Unit

	Valve Surgery Group		Coronary Surgery Group		Total sample	
	Dental assessment at InCor n (%)	No InCor dental assessment n (%)	Dental assessment InCor n (%)	No InCor dental assessment n (%)	Dental assessment at InCor InCor n (%)	No InCor dental assessment n (%)
With infection	15 (8.3)*	70 (11.9)*	2 (5.7)*	50 (10.6)*	17 (7.9)*	120 (11.3)*
No infection	165 (91.7)*	522 (88.1)*	33 (94.3)*	422 (89.4)*	198 (92.1)*	939 (88.7)*
Total	180 (23.5)#	592 (76.5)#	35 (6.9)#	472 (93.1)#	215 (16.9)#	1059 (83.1)#
p value	0.1793		0.5627		0.1395	

* Column percentage;

# row percentage.

Patients who underwent prior dental assessment had a shorter length of stay than those without evaluation in both the VSG (p=0.0187) and CSG (p=0.0317) (Table 6).

**Table 6:** Length of stay according to the presence or absence of prior dental assessment

Dental assessment	Valve Surgery Group	Coronary Surgery Group	Total sample
Dental assessment	16 (13)	12 (9)	15 (13)
No dental assessment	18 (16)	14 (12)	16 (14)
p value	0.0187	0.0317	0.1648

## DISCUSSION

Among the studies identified in the literature, only the study by Hakeberg et al.,^(13)^ conducted in Sweden, used an approach similar to that of the present study. In that prospective study of 253 patients undergoing valve surgery, the authors concluded that dental treatment did not decrease the rate of immediate infections after valve surgery. However, they noted that the long-term consequences of prior dental treatment remained unclear. The sample size in that study was much smaller than ours; in addition, good oral health was reported in the studied population, largely reflecting the country's national dental health insurance system, which covers dental care—a context that does not apply to the Brazilian population.

According to epidemiological data collected by the InCor Dentistry Unit between 1995 and 2004, only 19.5% of 1,000 patients preparing for heart valve surgery had good oral health. Good oral health was defined as the absence of periodontal disease and the presence of at least 20 teeth, indicating that poor oral health was prevalent in this patient population. Although this condition may not be a risk factor for immediate post-surgical cardiac infections, it should not be neglected.^(13,15)^ Although bacteremia is not the only factor associated with infection, the removal of acute and/or chronic oral infectious foci may help restore oral health by reducing bacterial load and the frequency of bacteremia, thereby potentially contributing to the prevention of infective endocarditis and ventilator-associated pneumonia by supporting oral microbiota balance.^(9,11,15,16,18,21)^

The most prevalent microorganisms in the infection processes affecting the study sample belonged to the genus Staphylococcus, which is frequently associated with SSIs^(22)^ and is therefore less commonly found in the oral cavity. Sands et al.^(10)^ evaluated the oral biofilm microbiota of patients receiving mechanical ventilation and observed Staphylococcus aureus and Pseudomonas aeruginosa in 32.7% of evaluated patients, suggesting that dental plaque may represent a potential source of pathogens for ventilator-associated pneumonia; this condition is associated with changes in the oral microbiota due to factors such as reduced immune function and patient frailty.

Patients who underwent dental assessment had shorter hospital stays in both groups. This finding has not been reported consistently in previous studies. In a study conducted in Brazil, De Souza et al.^(15)^ showed that patients who underwent dental treatment before surgery had a longer hospital stay than those who did not undergo dental treatment (21.5 *versus* 14 days). Because the study group had characteristics similar to those of the present study population, their oral health status may also have been similar. However, the study by De Souza et al.^(15)^ included 481 patients, a smaller sample than that evaluated in the present study. Thus, because prolonged hospitalization is associated with higher hospital costs,^(4)^ this finding is relevant both clinically and economically.

Several studies have evaluated the risks of dental treatment in patients scheduled for cardiac surgery.^(14–18)^ For example, Nakamura et al.^(18)^ evaluated the risk factors associated with dental treatment before cardiac surgery, including extractions, in patients scheduled for valve surgery. The authors concluded that dental treatment had no significant impact on postoperative complication rates and could therefore be safely performed before valve surgery. Nevertheless, individual assessment remains essential for planning and determining the appropriate timing of dental treatment.^(13–18)^

The infection rate observed in the present study was higher than that reported in high-income settings but compatible with rates reported in some low- and middle-income settings.^(1–3,6,22)^ In a study of 5,158 patients undergoing cardiac surgery in the United States, Gelijns et al.^(2)^ observed a 4.6% prevalence of post-surgical infection. In China, Jiang et al.^(3)^ found that 7.8% of 1,606 patients developed post-surgical infection, whereas in Brazil, Soares et al.^(1)^ found that 18.1% of 204 patients developed post-surgical infection; the latter study had a smaller sample than the present study, which may partly explain the difference in results.

Most surgeries performed in the present study were valve surgeries, which differs from previous studies reporting a higher prevalence of coronary artery bypass grafting.^(1,5,23)^ This finding may be related to the characteristics of the hospital, which is a tertiary university hospital focused on highly complex cases.

Male sex, which was more prevalent in the CSG, is considered a risk factor for hypercholesterolemia and atherosclerotic disease.^(23,24)^ Hypertension, diabetes mellitus, and dyslipidemia are also risk factors for atherosclerotic disease,^(23,24)^ and these comorbidities were prevalent in the study sample. Female sex, which was predominant in the VSG, has been associated in previous studies with a greater predisposition to some valvular heart diseases.^(12)^ Some comorbidities, such as hypertension, may be associated with valvular heart diseases,^(12,25)^ as observed in the present sample. Although no association between comorbidities and infection was observed in the present study, some comorbidities, such as diabetes, may be associated with a higher occurrence of postoperative infection by impairing healing, contributing to vascular complications, and reducing host defense mechanisms.^(3,5,6,26)^

The most frequently identified microorganisms were closely related to the most frequent infection diagnoses observed in both groups, consistent with the literature.^(2,27,28)^ The SSI rate was within the range reported in Brazilian studies but remained above ideal levels. These infections are considered preventable because they are associated with maintenance of the aseptic chain from patient preparation to the surgical team's intraoperative practices.^(22)^ In high-income countries, SSI rates range from 1.2% to 5.2%, whereas in low- and middle-income countries, they may reach 11.8%.^(22)^ In Brazilian studies, Andrade et al.^(5)^ and Soares et al.^(1)^ reported SSI rates of 8.3% and 6.6%, respectively. By contrast, pneumonia is the most prevalent postoperative infection after cardiac surgery in high-income countries,^(2–4,27,29)^ as reported by Gelijns et al.,^(2)^ in whose study 2.38% of patients developed pneumonia.

Previous studies^(2–5,22)^ show that prolonged hospitalization and death are associated with infection, as observed in the evaluated sample. Greco et al.^(4)^ concluded that major HAIs increase hospitalization by an average of 2 weeks, and Jiang et al.^(3)^ reported mortality rates of 16.8% in infected patients and 2.7% in non-infected patients.

### Study limitations

First, the retrospective design, based on medical records, was subject to information bias and did not allow control for undocumented confounding variables, such as unrecorded periodontal disease severity or antibiotic use for other indications. Second, the single-center design at InCor-HCFMUSP may limit the generalizability of the findings to other hospital settings, especially those with different epidemiological profiles and resources. Third, variability in dental evaluation protocols between surgical groups—routine in the VSG and complementary in the CSG—introduced a potential source of indication bias because the groups were not completely comparable. Finally, the absence of standardized and quantifiable clinical oral-health parameters, such as plaque indices, gingival bleeding, and number of missing teeth, prevented a more robust analysis of which specific oral conditions were associated with higher risk, restricting the analysis to an "evaluated versus not evaluated" dichotomy. These limitations should be considered when interpreting the results.

### Future directions and clinical implications

These findings suggest important directions for future research and clinical practice. Prospective multicenter studies with larger samples are needed to clarify the relationship between oral health and post-surgical infections. Future investigations should focus on developing and validating standardized dental clearance protocols for cardiac surgery based on objective and measurable criteria. Furthermore, evaluating the impact of specific dental interventions, such as periodontal scaling and extractions, on outcomes such as length of hospital stay and incidence of ventilator-associated pneumonia would be valuable. From a clinical and translational perspective, this study supports further consideration of systematic multidisciplinary collaboration between dental and cardiology teams. Although this study did not demonstrate a statistically significant reduction in total infections, the descriptive finding that 89.8% of patients in the dental assessment group remained infection-free and the significant reduction in length of hospital stay among evaluated patients (p=0.0187 in the VSG and p=0.0317 in the CSG) may have clinical and economic relevance. These data support further evaluation of preoperative dental assessment as part of multidisciplinary preparation for elective cardiac surgery, especially in settings where poor oral health is prevalent, and may encourage integration between specialties to optimize perioperative outcomes.

## CONCLUSION

There is no significant relationship between pre-cardiac surgery oral health and chronic venous insufficiency; however, it should be considered that poor oral health can be a risk factor for the development of ventilator-associated pneumonia and late infections such as endocarditis. Therefore, dental treatment aimed at removing foci of infection is important. The prevalence of chronic venous insufficiency was low, with no difference observed between the groups studied. Further studies should be conducted to clearly establish the relationships between dental treatment and chronic venous insufficiency in cardiovascular surgeries.

## Data Availability

The underlying content is contained within the manuscript.
